# Antibiotic Resistance Related to Biofilm Formation in *Klebsiella pneumoniae*

**DOI:** 10.3390/pathogens3030743

**Published:** 2014-09-05

**Authors:** Claudia Vuotto, Francesca Longo, Maria Pia Balice, Gianfranco Donelli, Pietro E. Varaldo

**Affiliations:** 1 Microbial Biofilm Laboratory, IRCCS Fondazione Santa Lucia, Rome 00179, Italy; E-Mails: f-longo@hotmail.it (F.L.); g.donelli@hsantalucia.it (G.D.); 2 Clinical Microbiology Laboratory, IRCCS Fondazione Santa Lucia, Rome 00179, Italy; E-Mail: mp.balice@hsantalucia.it; 3 Department of Biomedical Sciences and Public Health, Section of Microbiology, Polytechnic University of Marche, Ancona 60126, Italy; E-Mail: pe.varaldo@univpm.it

**Keywords:** *Klebsiella pneumonia*, biofilm, antibiotic resistance

## Abstract

The Gram-negative opportunistic pathogen, *Klebsiella pneumoniae*, is responsible for causing a spectrum of community-acquired and nosocomial infections and typically infects patients with indwelling medical devices, especially urinary catheters, on which this microorganism is able to grow as a biofilm. The increasingly frequent acquisition of antibiotic resistance by *K. pneumoniae* strains has given rise to a global spread of this multidrug-resistant pathogen, mostly at the hospital level. This scenario is exacerbated when it is noted that intrinsic resistance to antimicrobial agents dramatically increases when *K. pneumoniae* strains grow as a biofilm. This review will summarize the findings about the antibiotic resistance related to biofilm formation in *K. pneumoniae*.

## 1. Introduction

*Klebsiella pneumoniae* was isolated for the first time in 1882 by Friedlander from the lungs of patients who died after pneumonia. This encapsulated bacterium, initially named Friedlander’s bacillus, was renamed *Klebsiella* in 1886. Later, it was described as a saprophyte microorganism, not only colonizing the human gastrointestinal tract, skin and nasopharynx, but also able to cause urinary and biliary tract infections, osteomyelitis and bacteremia [1,2,3].

The virulence factors playing an important role in the severity of *K. pneumoniae* infections are capsular polysaccharides, type 1 and type 3 pili, factors involved in aggregative adhesions and siderophores [3,4,5,6], with those studied in greater depth being capsular polysaccharides and type 1 and type 3 pili.

Capsules, whose subunits can be classified into 77 serological types [7], are essential to the virulence of *Klebsiella* [8]. The capsular material, forming fibrillous structures that cover the bacterial surface [9], protects the bacterium from phagocytosis, on the one hand [10], and prevents killing of the bacteria by bactericidal serum factors, on the other [11].

Type 1 and type 3 pili [12] (otherwise known as fimbriae), instead, are non-flagellar, filamentous fimbrial adhesins, often detected on the bacterial surface, that consist of polymeric globular protein subunits (pilin) [13]. On the basis of their ability to agglutinate erythrocytes and depending on whether the reaction is inhibited by d-mannose, these adhesins are designated as mannose-sensitive (MSHA) or mannose-resistant hemagglutinins (MRHA), respectively [14]. Type 1 fimbriae are encoded by an operon (*fim*) containing all of the genes required for the fimbrial structure and assembly, with assembly occurring via the chaperone-usher pathway [15]. Type 1 fimbriae in *K. pneumoniae* are regulated via phase variation in a similar way to the regulation of type 1 fimbriae in *E. coli* [16,17]. Alcántar-Curiel and colleagues demonstrated that the *fim* operon was found in 100% of 69 *K. pneumoniae* isolates and that type 1 fimbriae were detected in 96% of these strains [18]. Conversely, the type 3 fimbriae are encoded by the *mrk* operon and are predicted to be assembled via a chaperone-usher pathway, too, with the *mrk* gene cluster being chromosome or plasmid borne [19,20,21].

Due to its high pathogenicity, *K. pneumoniae*, in the pre-antibiotic era, was considered as an important causative agent of community-acquired (CA) infections, including a severe form of pneumonia, especially in alcoholics and in diabetic patients. In recent years, while CA pneumonia due to this pathogen has become rare, novel manifestations of *K. pneumoniae* CA infections, including liver abscess complicated by endophthalmitis, different metastatic infections [22], often caused by highly virulent strains of specific serotypes, such as K1 [23], as well as urinary tract infections [24], have been described.

The greater adhesiveness and presumably also the invasiveness of strains may play an important role in the recurrent infections, *K. pneumoniae* strains being able to persist despite appropriate antibiotic treatment [25]. However, unlike the adhesion ability, the invasive capacity of *K. pneumoniae* to cause liver infections [26,27] and urinary tract infections [28] is still controversial and requires further study.

In contrast, starting from the early 1970s, *K. pneumoniae* epidemiology and its spectrum of infections significantly changed when this microorganism was established in the hospital environment and became a leading cause of nosocomial infections, particularly in developed Western countries. In fact, its considerable efficiency of colonization, accompanied by acquired resistance to antibiotics, has enabled *K. pneumoniae* to persist and spread rapidly in healthcare settings, the most common healthcare-associated infections caused by this agent involving the urinary tract, wounds, lungs, abdominal cavity, intra-vascular devices, surgical sites, soft tissues and subsequent bacteremia [3,29,30,31].

Klebsiella is second only to *Escherichia coli* in nosocomial Gram-negative bacteremia [32], as well as in urinary tract infections (UTIs), affecting catheterized patients (16% and 70%, respectively) [33]. In fact, *K. pneumoniae* has been reported as a prominent cause of infections in individuals with indwelling urinary catheters [34,35].

Of interest, a high incidence of *K. pneumoniae* in UTIs (from 6% to 17%) was reported in previous studies carried out in specific groups of patients at risk, e.g., patients with diabetes mellitus or with neuropathic bladders [36,37].

As concerns the bacteremia associated with intravascular catheters, an epidemiological study on bloodstream infections carried out in Israel revealed that *Staphylococcus aureus* was the most common species (30%), followed by *K. pneumoniae* (10%) [38].

In general, a cohort study indicated that the majority of infections associated with different medical devices, including both urinary and intravascular catheters, was caused by *K. pneumoniae* followed by staphylococcal biofilms, and a high percentage (about 90%) of biofilm-producing bacterial isolates causing infection were multidrug resistant [39].

In 2013, the incidence of *K. pneumoniae* clinical infections was estimated in the United States to be higher in long-term acute care hospitals, compared to short-stay hospital intensive care units [40].

In a prospective study on hospital-acquired infections carried out in Rome in the period January, 2002–December, 2004, *K. pneumoniae* was reported as the second most frequent Gram-negative species (11%) after *Pseudomonas* (25%) [41]. In a countrywide cross-sectional survey carried out in collaboration with twenty-five large clinical microbiology laboratories from 23 Italian cities, Klebsiella pneumoniae carbapenemase (KPC)-producing *K. pneumoniae* (KPC-KP) were revealed to majorly contribute to the epidemic dissemination of carbapenem-resistant *Enterobacteriaceae*, their spreading being mostly sustained by strains of clonal complex 258 (ST-258 producing KPC-2 or KPC-3 and ST-512 producing KPC-3) [42].

In a recent meta-analysis covering studies (2000–2010) on Gram-negative wound infections in hospitalized adult burn patients, *K. pneumoniae* has been reported as one of the most common Gram-negative pathogens, after *P. aeruginosa* [43].

## 2. Antibiotic Resistance

Although *K. pneumoniae* possesses only moderate amounts of chromosomal penicillinases, it is a well-known “collector” of multidrug resistance plasmids that commonly encoded resistance to aminoglycosides, till the end of 1980s, while, later, encoding extended-spectrum β-lactamases (ESBLs), mostly Temoniera (TEMs) and Sulfhydryl variable (SHVs) active against last generation cephalosporins, as well as a variety of genes conferring resistance to drugs other than β-lactams [3,44].

The acquisition of these plasmids and the occurrence of chromosomal mutations that confer resistance to fluoroquinolones often makes the treatment of *K. pneumoniae* healthcare-associated infections possible only by using carbapenems as “last-line of defense” antibiotics [45,46,47].

Unfortunately, from the early 2000s, multidrug-resistant (MDR) *K. pneumoniae* strains started to produce also “carbapenemases” encoded by transmissible plasmids and rapidly disseminated within both acute hospitals and long-term care facilities. Later, other enterobacterial species, including *E. coli*, acquired carbapenemase genes, thus suggesting that *K. pneumoniae* may have acted as a pool of β-lactamases [48].

Over the past decade, carbapenemases of Classes A, B and D, which are β-lactamases able to efficiently hydrolyze penicillins, cephalosporins, monobactams, carbapenems and β-lactamase inhibitors, have progressively disseminated among *Enterobacteriaceae* [49]. The Class A KPCs, firstly discovered in the USA in 1996, are the most worrying carbapenemases for their spreading across countries and continents, even if their expansion is dependent on the geographical area [50,51]. Furthermore, the zinc-dependent Class B metallo-β-lactamases (MβLs), mainly represented by the Verona integron-encoded metallo-β-lactamase (VIM), imipenemase metallo-β-lactamase (IMP) and New Delhi metallo-β-lactamase (NDM) types, are encoded on highly transmissible plasmids that spread rapidly between bacteria, rather than relying on clonal proliferation. In particular, NDM-1, originated in Asia, was found in almost every continent within one year from its emergence in India [52]. The picture is completed by the plasmid-expressed Class D carbapenemases of the oxacillinase-48 (OXA-48) type [48,53,54,55].

KPC enzyme-producing *K. pneumoniae* is generally susceptible to few antibiotics, and it is associated with a high mortality rate among patients with bloodstream infections. In fact, many of these strains are susceptible to colistin, tigecycline and one or more aminoglycosides, but some of them are resistant even to these drugs [56].

## 3. Biofilm

*K. pneumoniae* was reported to be able to grow *in vitro* as a biofilm since the end of the 1980s [57], but clear evidence of an *in vivo* biofilm was provided only in 1992 by Reid and coworkers, who investigated by scanning electron microscopy some bladder epithelial cells of a spinal cord injured patient with an asymptomatic urinary tract infection caused by *K. pneumoniae* [58].

Later, *in vitro* studies have demonstrated that about 40% of *K. pneumoniae* isolated not only from urine, but also from sputum, blood and wound swabs, were able to produce biofilm [59], as well as that about 63% of *K. pneumoniae* isolates from urine samples of catheterized patients suffering from UTIs were positive for *in vitro* biofilm production [33]. Recently, also a high rate of *K. pneumoniae* strains isolated from endotracheal tubes (ETT) of patients affected by ventilator-associated pneumonia (VAP) were reported to be able to form an *in vitro* biofilm [39].

**Figure 1:**
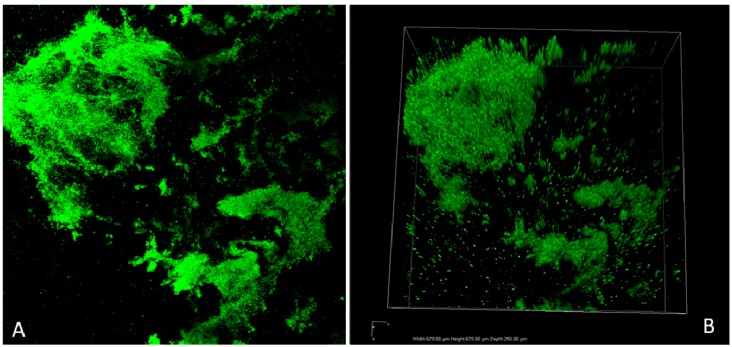
Bidimensional (**A**) and three-dimensional (**B**) images of a *K. pneumoniae in vitro* biofilm obtained by Confocal Laser Scanning Microscopy (CLSM) on different areas of a biofilm-covered glass coverslip. SYTO ® 9 green fluorescent nucleic acid stain has been used to detect both live and dead bacteria (Life Technologies, Monza (MB) , Italy).

Biofilm formation on abiotic surfaces was shown to be more consistent at 40 °C than 35 °C, using atomic force and high-vacuum SEM [60]. The ability of *K. pneumoniae* clinical strains to adhere and form biofilm *in vitro* was recently investigated by field emission scanning electron microscopy (FESEM) [61] and by confocal laser scanning microscopy (Figure 1).

### 3.1. Virulence and Biofilm Formation

Type 1 or type 3 fimbriae, as well as the capsule and the LPS are the virulence factors mostly involved in the ability of *K. pneumoniae* to grow as biofilm.

Type 3 fimbriae have been demonstrated to be the major appendages that mediate the formation of biofilms on biotic and abiotic surfaces and the attachment to endothelial and bladder epithelial cell lines [62,63,64,65,66,67]. In particular, growth of *K. pneumoniae* on abiotic surfaces is facilitated by the MrkA type 3 fimbrial protein, whereas growth on surfaces coated with a human extracellular matrix (HECM) requires the presence of the type 3 fimbrial adhesin MrkD [62,64].

Recent investigations have verified that type 3 fimbrial gene expression is regulated, at least in part, by the intracellular levels of cyclic di-GMP [68].

Type 3 fimbriae have been confirmed by Murphy and colleagues (2013) to be a very important colonization factor in biofilm-mediated infections associated with catheter-associated urinary tract infections (CAUTIs) by obtaining mutants lacking the ability to produce type 1 or type 3 fimbriae or a combined double mutant. These mutants were impaired in colonization and had subsequent persistence under specific experimental conditions [69].

Alcántar-Curiel and colleagues demonstrated that, among the 69 examined *K. pneumoniae* isolates, 55 were able to produce biofilm and all of them contained *mrkA*, but only 57% of them produced MR/K fimbriae [18]. The same group also revealed that 96% of 69 *K. pneumoniae* isolates harbored the *ecpABCDE* operon homolog of the operon encoding the *E. coli* adhesive structure common pilus (ECP), with 94% of them producing ECP during adhesion to cultured epithelial cells and 8% during the formation of biofilms on glass. These results suggest that ECP also seems to be required for biofilm formation, at least *in vitro* [18].

Regarding the involvement of capsule and LPS in *K. pneumoniae* biofilm formation, it has been proven that both of them contribute to the structure of biofilm communities of *K. pneumoniae*. In fact, gene disruption and microscopic analyses showed that LPS is involved in the initial adhesion on abiotic surfaces and that the capsule is necessary for a proper initial coverage of substratum and construction of mature biofilm architecture [70].

Furthermore, *treC* and *sugE* genes have been demonstrated to affect biofilm formation by modulating CPS production [71].

However, conflicting results on the involvement of CPS production in biofilm formation arise from a paper of Huang and coworkers (2014). In fact, the authors found that two knockout mutants, the first one with the entire gene cluster responsible for biosynthesis of the extracellular polysaccharide capsule deleted and the other one with the capsule export subsystem deleted, have lower amounts of capsule, but produce greater amounts of biofilm [72].

### 3.2. Involvement in Mixed Biofilms

The first investigation of mixed microbial populations including *K. pneumoniae* dates back to 1991. Siebel and colleagues demonstrated the ability of *K. pneumoniae* to form *in vitro* a dual-species biofilm together with *P. aeruginosa*. Authors determined that, although the *K. pneumoniae* specific cellular growth rate was five times that of *P. aeruginosa*, it did not dominate the microbial population, with results indicating that neither the specific cellular product formation rate nor the glucose-oxygen stoichiometric ratio of *K. pneumoniae* or *P. aeruginosa* when grown together were affected by the presence of the other species [73].

Later, *K. pneumoniae* was shown to form a biofilm more successfully in a mixture than in isolation with an increased resistance to disinfection [74].

In the same year, Stickler and colleagues, by using a model of a catheterized bladder, firstly investigated the possible role of *K. pneumoniae* and other uropathogens in the development of crystalline biofilm on catheter surfaces, demonstrating that this microorganism is not able to raise the urinary pH and, thus, to contribute to the crystalline biofilm formation [75].

In 2007, the same research team analyzed 106 biofilms samples developed on urinary catheters, finding that *K. pneumoniae* was able to form a mixed biofilm with *Proteus mirabilis*, when *E. coli*, *Morganella morganii* or *Enterobacter cloacae* were also present [76].

The ability of *K. pneumoniae* to form a mixed-species biofilm *in vitro* with *P. aeruginosa* and *Pseudomonas protegens* was confirmed by adding a fluorescent tag to each species in order to determine the abundance and spatial localization of each of them within the biofilm. The development of the mixed-species biofilm was delayed 1–2 days compared with the single-species biofilms, and the composition and spatial organization of the mixed-species biofilm changed along the flow cell channel. Furthermore, the mixed-species biofilm resulted in being more resistant to sodium dodecyl sulfate and tobramycin with respect to the single-species biofilms [77].

Recent investigations on 35 ETTs obtained from 26 neonates with mechanical ventilation demonstrated that *K. pneumoniae*, together with *Pseudomonas* and *Streptococcus*, was the most common bacteria isolated from ETT-bacterial biofilms, and it was hypothesized that there were interactions among these species in the biofilm [78].

**Figure 2:**
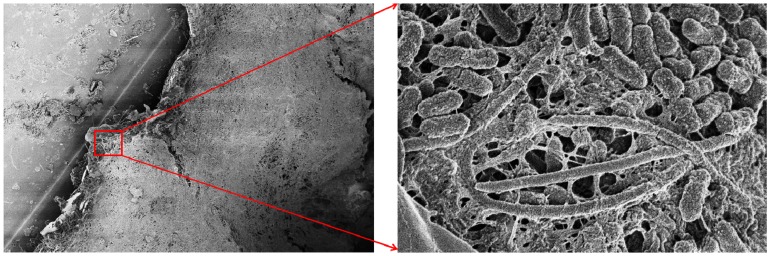
FESEM micrographs (**a** = 2000×; **b** = 25,000×) of a polymicrobial biofilm grown in the lumen of a urinary catheter removed from a patient recovered at the research hospital for neuromotor rehabilitation, Fondazione Santa Lucia in Rome. The species identified by culture methods were *Klebsiella pneumonia* and *Candida albicans*.

Our recent findings demonstrated that *K. pneumoniae* is able to form a multi-species biofilm together with *Candida albicans* within a urinary catheter removed from a patient hospitalized at the neuromotor rehabilitation hospital, Fondazione Santa Lucia in Rome, Italy (Figure 2).

## 4. Antibiotic Resistance of Biofilm-Growing Strains

The response of *K. pneumoniae* biofilm to different antimicrobial agents has been investigated in several studies in the last decade.

One of the first studies that addressed the issue of the penetration of antimicrobials through *K. pneumoniae* biofilm was in 2000, when Anderl and co-workers used an *in vitro* model system to evaluate the effect of ampicillin and ciprofloxacin on *K. pneumoniae* biofilm developed on microporous membranes with agar nutrient medium. *K. pneumoniae* biofilms resisted killing during prolonged exposure to both antibiotics. The authors directly measured the antibiotics’ diffusion, demonstrating that ampicillin did not penetrate wild-type *K. pneumoniae* biofilms, whereas ciprofloxacin and a nonreactive tracer (chloride ion) penetrated the biofilms quickly. Ampicillin was able to penetrate biofilms only when formed by a β-lactamase-deficient mutant, thus demonstrating that the increased resistance of both wild-type and mutant *K. pneumonia* biofilm to ampicillin and ciprofloxacin could not be attributed to slow diffusion [79].

The penetration of ampicillin and ciprofloxacin through *K. pneumoniae* biofilms was then confirmed using transmission electron microscopy (TEM). The authors visualized cells in biofilm after antibiotic exposure, identifying those regions of the biofilm contained on an agar plate that were able to reach 10-fold minimum inhibitory concentration (MIC) with ciprofloxacin or ampicillin [80].

This topic was further addressed one year later by Andrel and colleagues, assuming that the already observed mechanism might be due to the presence, in internal zones of biofilm with nutrient limitations, of stationary-phase bacteria that become tolerant to ampicillin and ciprofloxacin, as observed in free-floating bacteria [81].

A number of other antibiotics, including piperacillin, piperacillin-tazobactam, cefoperazone, ceftazidime, cefepime, meropenem, ciprofloxacin, netilmicin and amikacin, were also evaluated on *K. pneumoniae* biofilm, confirming that adherent bacterial populations exhibited reduced antimicrobial susceptibility with respect to their planktonic counterpart [82].

On the contrary, in 2009, tetracycline and chloramphenicol at a five-fold MIC were demonstrated to affect *K. pneumoniae* biofilms, even if to a different extent (*p* < 0.05 and *p* < 0.01, respectively) [83], and their inhibitory effects could be explained by the fact that both of these antibiotics are protein synthesis inhibitors, which act on the bacterial ribosome [84,85].

The higher resistance of *K. pneumoniae* biofilm to ciprofloxacin, amikacin and piperacillin was confirmed by testing them on different phases relevant for biofilm formation, including planktonic cells at mid-log phase, planktonic cells at stationary phase, adherent monolayers and mature biofilms. *K. pneumoniae* in a biofilm growth mode resulted in being more resistant to all antibiotics. The effect of amikacin and ciprofloxacin on young and older biofilms, at the highest achievable serum concentrations, was also examined, observing that amikacin was able to eradicate the young biofilms, but became completely ineffective when biofilm increased in age. A possible explanation for the increasing resistance during the time of growth was given by calcofluor staining, an enhanced production of exopolysaccharide in older biofilms being observed [86].

Bellifa and coworkers investigated the response of biofilm-growing *K. pneumoniae* strains isolated from medical devices to gentamicin, cefotaxime and ciprofloxacin, detecting that isolates were at least 10–25-times more resistant when grown as a biofilm than in the planktonic form [87].

Recently, differences in the antibiotic resistance of biofilm-growing *K. pneumoniae* strains to gentamicin, depending on the resistance or susceptibility of the planktonic-growing isolates to this antibiotic, have been observed. In fact, gentamicin-resistant isolates dramatically increased their resistance when grown as a biofilm (up to 234-fold), whereas gentamicin-susceptible isolates preserved their susceptibility also in biofilm, thus supporting the use of this antibiotic to successfully treat gentamicin-susceptible biofilm-growing *K. pneumoniae* strains [88].

The efficacy of amikacin has been evaluated also by developing a biofilm model of *K. pneumoniae* B5055, mimicking *in vivo* biofilm system. By using the BacLight viability staining kit, the antibiotic was effective against younger biofilm, but ineffective against older biofilm, possibly due to the heterogeneity and thickness of the biofilm itself [89].

Contrariwise, imipenem recently has shown a potent activity against established *K. pneumoniae* biofilms under both static and flow conditions *in vitro* and *in vivo*, by using a rabbit ear wound model [90].

## 5. Correlation between Biofilm and Antibiotic Resistance

To date, it has been demonstrated that some correlations exist between antibiotic resistance and the biofilm-forming ability of *K. pneumoniae* strains.

For instance, the ability of 150 *K. pneumoniae* strains, isolated from sputum and urine, to form biofilm exhibited a significant association with their ESBL production. In fact, among the 44.7% biofilm formers, 45.3% of them produced ESBLs [59].

Later, a NDM-1 carrying a *K. pneumoniae* isolate has been demonstrated to be the most virulent in the murine sepsis model and the stronger biofilm producer, as tested with the Calgary device method, with respect to the non-NDM-1 carrying isolates, but there was no clear correlation with *in vitro* virulence factors, such as biofilm formation ability or killing in *Caenorhabditis elegans* [91].

In 2012, a prospective analysis revealed that 80% of the biofilm-producing strains collected from 100 urine samples from catheterized patients with symptoms of UTI over a period of six months, exhibited the MDR phenotype. In particular, biofilm-positive isolates showed 93.3%, 83.3%, 73.3% and 80% resistance to nalidixic acid, ampicillin, cefotaxime and co-trimoxazole, respectively, compared to the 70%, 60%, 35% and 60% resistance shown by biofilm non-producers for the respective antibiotics [92].

Afterwards, Sanchez and coworkers confirmed the propensity of MDR *K. pneumoniae* strains to form a richer biofilm with respect to the susceptible ones, with special reference to those resistant to cephalosporins [93].

The link between antibiotic resistance and biofilm formation has been also examined by growing *K. pneumoniae* strains under antibiotic pressure, mostly with a sub-inhibitory concentration of antimicrobials. Hennequin and colleagues monitored the bactericidal effect of cefotaxime (MIC 516 mg/L) and ofloxacin (MIC 2 mg/L) on CTX-M-15-producing *K. pneumoniae*. While in the presence of sub-MICs of ofloxacin, the biomass decreased in inverse proportion to the antibiotic concentrations; in the presence of cefotaxime at sub-MIC concentrations, the biofilm formation enhanced [94].

Finally, looking more specifically at antibiotic resistance genes responsible for this correlation, *AmpR*, a regulator of *K. pneumoniae* virulence, particularly regulating a cephalosporins resistance gene (*DHA-1*) carried on a plasmid, has been demonstrated to modulate biofilm formation and type 3 fimbrial gene expression, as well as the adhesion to HT-29 intestinal epithelial cells [95].

## 6. Conclusions

The opportunistic pathogen, *K. pneumoniae*, can give rise to severe diseases, typically nosocomial infections, such as septicemia, pneumonia, UTI and soft tissue infection. *Klebsiella* infections are often considered as a paradigm of hospital-acquired infections. The indiscriminate use of antibiotics has revealed a considerable increase in outbreaks caused by microorganisms resistant to antimicrobial drugs, such as KPC-producing *K. pneumoniae*.

Nosocomial *Klebsiella* infections continue to be a heavy burden on the economy and on the life expectancy of patients in developed countries. Thus, further progress in the prevention of hospital-acquired infections will require new approaches to infection control.

The increasing evidence on the ability of *K. pneumoniae* to form biofilm, mostly on medical devices and the recent data supporting the correlation of such a behavior with the antibiotic resistance acquisition should alert even more regarding the hazard of this pathogen in hospital settings.

The exploration of these virulence factors and the study of new mechanisms to control them could be an important way to counteract *K. pneumoniae* nosocomial infections. In particular, the biofilm mode of growth makes bacteria up to 1,000-times more resistant to antibiotic therapy. In *K. pneumoniae*, many studies were performed in order to better highlight the mechanisms underlying this resistance, demonstrating that the limitation of the penetration of antibiotic molecules through the biofilm matrix is not the main reason for the increasing resistance, but rather the slow growth rate in the center of biofilm is. In any case, other mechanisms are involved, and further studies are requested as a future challenge to elaborate new concepts in the preventive measures against nosocomial *K. pneumoniae* infections.
